# A Review of the Phytochemistry and Pharmacological Activities of Raphani Semen

**DOI:** 10.1155/2013/636194

**Published:** 2013-07-08

**Authors:** Tung-Ting Sham, Ailsa Chui-Ying Yuen, Yam-Fung Ng, Chi-On Chan, Daniel Kam-Wah Mok, Shun-Wan Chan

**Affiliations:** ^1^Department of Applied Biology and Chemical Technology, The Hong Kong Polytechnic University, Hong Kong; ^2^State Key Laboratory of Chinese Medicine and Molecular Pharmacology, 518057 Shenzhen, China; ^3^Food Safety and Technology Research Centre, Department of Applied Biology and Chemical Technology, The Hong Kong Polytechnic University, Hong Kong

## Abstract

The dried ripe seed of *Raphanus sativus* L., commonly known as radish seed (or Raphani Semen), is used as traditional Chinese medicine (TCM) to treat constipation, chronic tracheitis, and hypertension. The major active compounds in Raphani Semen are alkaloids, glucosinolates, brassinosteroids, and flavonoids. Fatty acids are its main nutritional contents. Raphani Semen has been demonstrated to have beneficial effects on hypertension, obesity, diabetes mellitus, constipation, and cough. So far, there is no report about the adverse/toxic effects of this herb on humans. However, Raphani Semen processed by roasting was reported to exhibit some adverse effects on mice. Additionally, erucic acid, the main fatty acid in Raphani Semen, was shown to enhance the toxicity of doxorubicin. Thus, Raphani Semen has a potential risk of causing toxicity and drug interaction. In summary, Raphani Semen is a valuable TCM herb with multiple pharmacological effects. More studies on Raphani Semen could help better understand its pharmacological mechanisms so as to provide clear scientific evidence to explain its traditional uses, to identify its therapeutic potential on other diseases, and to understand its possible harmful effects.

## 1. Introduction

**Figure 1:**
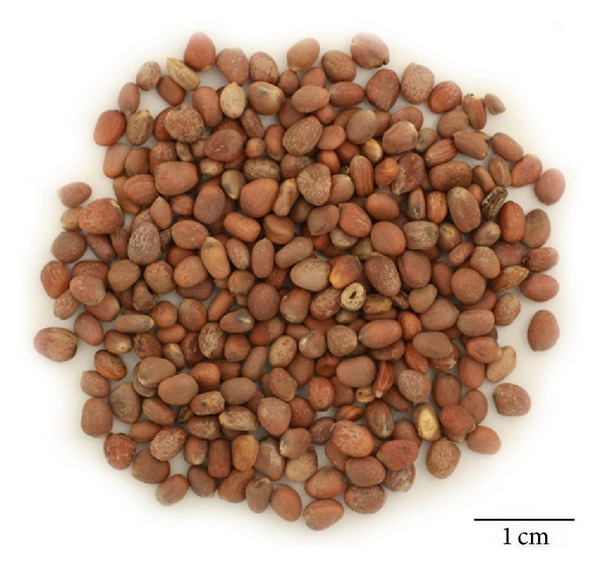
The dried seeds of *Raphanus sativus* L.

*Raphanus sativus *L. belongs to the family Brassicaceae (Cruciferae, an older name). It is known as Radish in English, Daikon in Japanese, and “Laifu” or “Luobo” in Chinese. With its high adaptive ability, high yield, and abundant nutritional value, *Raphanus sativus *L. has long been grown as a food crop worldwide, especially in China, Japan, Korea, and Southeast Asia [1]. The leaf, seed, and root of *Raphanus sativus *L. are claimed to have various medicinal uses [2], but only its dried ripe seed (Raphani Semen, Figure 1) is listed in Pharmacopoeia of the People's Republic of China (CP) [3]. Raphani Semen is commonly used in traditional Chinese medicine (TCM) for promoting digestion, relieving distention, directing “Qi” downward, and dissipating phlegm. Traditionally, it is used to treat food dyspeptic retention, distending pain in the epigastrium and abdomen, constipation, diarrhea and dysentery, panting, and cough with phlegm congestion clinically in combination with other TCM herbs [3, 4]. For example, Raphani Semen is one of the three important ingredients of San-Zi-Yang-Qin-Tang, which is a common TCM formula for relieving cough and asthma, dissipating phlegm, and promoting digestion [5].

Raphani Semen is usually called “Laifuzi” or “Luobozi” in Chinese. Its use in TCM can be traced back to ~900s A.D. in Ri-Hua-Zi-Zhu-Jia-Ben-Cao [3, 6]. The medical use of *Raphanus sativus* L. was even much earlier than that of Raphani Semen. It was recorded in Ming-Yi-Bie-Lu in 3-4 A.D [6]. Although the history of using Raphani Semen for medicinal purposes has more than a thousand years and it is one of the herbs documented in CP, there is no comprehensive review on the phytochemistry and pharmacology of Raphani Semen. 

With the advancement of analytical chemistry and pharmacology as well as the increase in the popularity of using natural products as an alternative medicine for treating chronic diseases such as cardiovascular diseases and cancer, research findings related to natural products expanded exponentially in recent decades [7–11]. This review summarized the recent scientific findings of Raphani Semen's phytochemistry and pharmacological activities, such as antihypertensive effect and other beneficial effects on digestive and respiratory systems, so as to provide a comprehensive overview of this herb.

## 2. Bioactive Chemical Constituents of Raphani Semen

Raphani Semen was found to have various classes of active compounds [2, 12], for instance, alkaloids, glucosinolates, brassinosteroids, and flavonoids. In fact, most of these phytochemicals have been shown to have different bioactivities (Tables 1, 3, and 4). 

### 2.1. Alkaloids

The total alkaloid content (in *terms of* sinapine thiocyanate equivalent) of Raphani Semen was found to range from 1.056% to 2.620% (weight percent) [13]. Water-soluble alkaloids of Raphani Semen demonstrated to have antihypertensive effects, which would be elucidated in the section of pharmacological effects in this review. Sinapine is a natural phenolic amine commonly found in the seeds of brassica (or cruciferous) plants [14, 15]. Sinapine is the major bioactive alkaloid [16], existing in the form of sinapine thiocyanate in Raphani Semen [17, 18]. In CP, the content of sinapine thiocyanate is used for the quality control of Raphani Semen. Previous studies have suggested that sinapine had antidiarrheal, anti-inflammatory [19], radioprotective [20, 21], neuroprotective [22], antioxidative [23], and antiaging effects [24]. Additionally, sinapine was shown to have potential in treating Alzheimer's disease by inhibiting the cerebral acetylcholinesterase activity [25, 26].

### 2.2. Glucosinolates and Isothiocyanates

Apart from sinapine, another group of well-known secondary product found in brassica plants is glucosinolates. Glucosinolates are stable compounds found in plant tissues. They carry no specific function. Generally, they would be transformed to other forms with higher bioactivity [69]. When intact cells of brassica plants are disrupted, glucosinolates are hydrolyzed by myrosinase or gut bacteria to form a series of products, such as isothiocyanate, thiocyanate, and nitriles [70, 71].

In dried Raphani Semen, total glucosinolate content was found to be 73.504 ± 0.980 *μ*mol/g. Among the glucosinolates in Raphani Semen, the content of glucoraphenin (40.338 ± 0.025 *μ*mol/g) is remarkably higher than glucoraphanin (0.762 ± 0.026 *μ*mol/g) [72]. Glucoraphanin was reported to be the modulator of hepatic cytochrome P450 and phase II conjugation activities in an *in vitro* study [29]. It may contribute to the chemopreventive activity of brassica vegetables.

**Figure 2:**
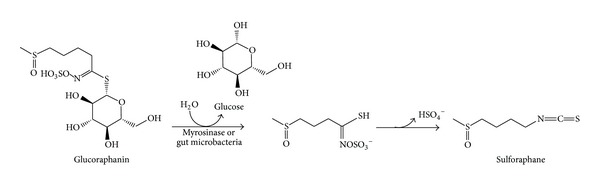
The hydrolysis process of sulforaphane from glucoraphanin by myrosinase or gut microbacteria.

Isothiocyanates, which are one type of the principal products of glucosinolates, were reported to be able to suppress tumour cell growth [73, 74]. Sulforaphane [27] and sulforaphene [42] are isothiocyanates derived from glucoraphanin and glucoraphenin, respectively. The content of sulforaphane and sulforaphene in Raphani Semen are 77.19–89.19 *μ*g/g [75] and 7 mg/g [30], respectively. The hydrolysis of glucoraphanin to sulforaphane [27] is shown in Figure 2. Sulforaphene was first identified via the investigation of antibacterial test with water extract of Raphani Semen [42] and later also found to possess growth inhibitory effect on human immortalized hepatocytes, to the similar extent as sulforaphane [39]. These two isothiocyanates were also found to have potentials in other chemoprevention of various cancers [1, 28, 31, 33–35, 37, 38, 40, 41]. In particular, sulforaphane was reported to be the potent inducer of phase II antioxidant enzymes [76, 77] implicated in carcinogen detoxification. Using molecular docking technique, sulforaphane was shown to bind to human serum albumins via nonpolar amino acids so as to deliver sulforaphane derived from glucoraphanin to target tissues [78]. The interaction of sulforaphane with serum albumins may give hints to the mechanism of actions of glucoraphanin or sulforaphane intake in cancer therapy. 

### 2.3. Brassinosteroids

Brassinosteroids represent a class of steroidal phytohormones showing high growth promoting activity in plants [79, 80]. Four kinds of brassinosteroids isolated from Raphani Semen are teasterone, 28-homoteasterone, brassinolide, and castasterone [81, 82]. A recent report provided the first evidence that natural brassinosteroids at micromolar concentrations could inhibit the growth of several human cancer cell lines without affecting the growth of normal cells [43]. The study suggested that castasterone showed significant growth inhibitory effect of some cancer cells but brassinolide did not. However, teasterone and castasterone represented biogenetic precursors of brassinolide [81, 83]. It is essential to isolate these compounds from the seeds to further identify their bioactivities.

### 2.4. Flavonoids

The total flavonoids (in *terms of* rutin content) in Raphani Semen were determined to be 0.60% [56]. Those flavonoids have been reported to have* in vitro* angiotensin converting enzyme (ACE) inhibitory activity through binding to the Zn^2+^ ion located at the active site of ACE [84].

## 3. Nutritional Contents in Raphani Semen

Radish is an important food crop; thus there are studies examining the nutritional ingredients of Raphani Semen. Fatty acids are the major nutritional composition of interests in Raphani Semen. Other nutritional components include minerals, vitamin, proteins, and polysaccharides.

### 3.1. Fatty Acids

Several studies have investigated the fatty acid composition in the oil of Raphani Semen [47–51]. The yield of its oil extracted by Soxhlet extraction was found to about 35% of its dry weight. The extraction yield could be up to 42.64 ± 1.36%, depending on the growing areas of Raphani Semen used. Many types of common fatty acids, including linolenic acid, erucic acid, oleic oil, eicosenoic acid, palmitic acid, stearic acid, arachidic acid, behenic acid, and 15-tetracosenoic acid, were identified using gas chromatography. Unsaturated fatty acids were found to account for over 80% of the total fatty acids. Linolenic acid, erucic acid, and oleic oil were the main fatty acids detected [47]. Oil of Raphani Semen was revealed to have significant antioxidative activity in the ferric reducing *antioxidant *power assay [47]. Additionally, linolenic acid was found to attenuate the formation of atherosclerotic plaques in rats fed with high-lipid diet through improving the antioxidation capacity of the body [59]. In a clinical trial, linolenic acid was revealed to be effective in improving the lipid profiles of 106 people with hyperlipidemia by taking soft capsules containing both *α*-linolenic acid and *γ*-linolenic acid [60]. Although the development of cardiovascular disease is multifactorial, hypercholesterolemia is believed to play a crucial role in its pathogenesis and progression [85]. Raphani Semen, which contains high content of linolenic acid, may have effects on reducing the risk of cardiovascular diseases.

### 3.2. Minerals

Minerals, which are trace elements, are directly involved in human metabolism. There is growing interest in the role of certain minerals in physiological processes [86]. Investigation on the mineral content in Raphani Semen is scarce. There was a study analyzing calcium, magnesium, zinc, and iron contents in the oil of Raphani Semen by using Inductively Coupled Plasma-Optical Emission Spectrometry [51]. Calcium was found to be the highest in content. The mineral contents of Raphani Semen are listed in Table 2.

### 3.3. Tocopherol Isomers

Tocopherols are one type of vitamin E homologues. Raphani Semen contains *α*-, *β*-, *γ*-, and *δ*-tocopherols [51], among which *γ*-tocopherol content is the highest (233 ± 6.63 *μ*g/g). Their contents and chemical structures are shown in Table 4. Tocopherols are important antioxidant compounds in oils. The oxidative stability of Raphani Semen oil mostly relied on the presence of tocopherols [67]. 

### 3.4. Proteins

Four purified antifungal proteins and one class of antifungal albumins were distinguished from Raphani Semen. They are *Raphanus sativus* antifungal proteins RAP-1, RAP-2 [55], Rs-AFP1 [52, 53], Rs-AFP2 [52, 54], and radish 2S storage albumins [52]. All purified antifungal proteins showed an inhibitory effect against *Botrytis cinerea *in the low ionic strength growth medium. On the other hand, RAP-1 exhibited stronger growth inhibitory activities against yeast *Candida albicans* and* Saccharomyces cerevisiae *than RAP-2. Rs-AFP1 and Rs-AFP2 are composed of highly basic 5 kDa polypeptides which are assembled in an oligomeric quaternary configuration with a high degree of specificity to filamentous fungi. The antifungal activity of Rs-AFP2 (IC_50_ values = 0.4–25 *μ*g/mL) is much more potent than Rs-AFP1 (IC_50_ values = 0.3–100 *μ*g/mL). Radish 2S storage albumins consist of at least five isoforms. They have significant growth inhibitory effect on various plant pathogenic fungi and some bacteria when assayed in the low ionic strength medium.

## 4. Pharmacological Activities of Raphani Semen

The extracts from Raphani Semen in different solvents were studied extensively. The pharmacological functions of Raphani Semen include antihypertensive effect, antilipase activity, improvement of the pathologic insulin resistance state, gastrointestinal effect, treatment of constipation, and antitussive therapeutic effect.

### 4.1. Antihypertensive Effect

The increase in blood pressure could raise the risks of myocardial infarction, heart failure, stroke, end-stage renal disease, peripheral vascular disease, and mortality from all causes [87]. In randomized clinical trials, antihypertensive therapy has also resulted in reductions in the incidence of stroke, myocardial infarction, and heart failure [88]. Thus, the development of treatment of hypertension attracted lots of research studies. The antihypertensive effect of Raphani Semen has been investigated in both preclinical and clinical settings in the past two decades.

The renin-angiotensin system is a hormone system that regulates blood pressure and water fluid balance, but it also participates in the pathophysiology of myocardial infarction, diabetic nephropathy, and congestive heart failure. ACE is one of the key therapeutic targets for the regulation of these pathophysiological developments. The ethanol extract of Raphani Semen was found to possess both *in vitro* ACE inhibitory and antioxidant activities. Thus, it could be used to treat hypertension, prevent or slow down the progression of cardiovascular and any free radical-related disorders. The presence of high phenolic and flavonoid contents in Raphani Semen extract would be a major contributor towards ACE inhibition through binding to Zn^2+^ at the active site of ACE [84]. Further *in vivo* studies on the effects of Raphani Semen on the renin-angiotensin system could give more insights on its anti-hypertensive effects.

The bioactive fractions, such as octenal, dibutyl phthalate, sinapine bisulfate, water-soluble alkaloids, and n-butanol extract, were found to have remarkable effects on lowering blood pressure [89, 90]. Systolic blood pressure of spontaneously hypertensive rats (SHR) measured by tail-cuff method decreased after treating the animals with high dosage of n-butanol extract of Raphani Semen [89]. The pharmacological mechanism of Raphani Semen's blood pressure lowering effect may be brought about by its vasodilation function to reduce the vascular resistance in the blood vessels [91]. Raphani Semen water extract exhibited blood pressure and heart rate reductions by mediating an atropine-sensitive pathway after being administrated to anaesthetized normotensive rat intravenously [92]. Such extract could also dose-dependently inhibit both atrial contractile force and rate of contraction in guinea-pig-isolated atria, where the inhibitory effect observed was found to be mediated through activation of muscarinic receptors [92].

Water-soluble alkaloids from Raphani Semen were demonstrated to have prominent hypotensive function by significantly enhancing serum nitric oxide (NO) content and cardiac nitric oxide synthase (NOS) activity in SHR [90]. Through decreasing serum malondialdehyde content and enhancing superoxide dismutase activity *in vivo*, Raphani Semen alkaloids are believed to have good cardiovascular protective effects from oxidative damage. In addition, choline, a metabolite from the degradation of sinapine in Raphani Semen extract in the digestive system, might activate the NO-NOS system [90], thereby increasing the synthesis of NO and resulting in vasodilation [9]. However, whether the anti-hypertensive effect of water-soluble alkaloids from Raphani Semen resulted from the increase in NO availability by directly enhancing NO production (stimulation of NOS activity), by indirectly reducing NO degradation (antioxidant properties), or by both pathways was currently unknown and warranted further investigations.

Raphani Semen has been used clinically in China. A proprietary drug called Qingxuanjianyia tablet is sold in China mainly containing concentrated Raphani Semen water extract in which sinapine is the key bioactive compound. It is used to treat hypertension. In a clinical trial, 160 hypertensive patients were randomly divided into four groups and orally administrated with Qingxuanjianyia tablets, nifedipine, captopril, or atenolol for 30 days [93]. The results indicated that intake of Qingxuanjianyia tablets could lower 82.5% of patients' blood pressure and ranked the third among the four tested medicines. There was no significant change on the heart rate as well as obvious side effects [93]. Another study with the same tablet compared to nifedipine showed similar results and suggested that Qingxuanjianyia tablet was suitable to mild and moderate hypertensive patients [94].

### 4.2. Antilipase Activity

Inhibition of pancreatic lipase is one of the potential approaches for the treatment of obesity. Pancreatic lipase is the main lipid-digesting enzyme to remove fatty acids from the *α* and *α*′ positions of dietary triglycerides to yield their lipolytic products and *β*-monoglyceride as well as long-chain saturated and polyunsaturated fatty acids [95]. The methanol extracts of Raphani Semen and 36 other Chinese medicinal herbs were tested for their effects on inhibiting porcine pancreatic lipase (PPL, type II) *in vitro*. Although Raphani Semen demonstrated a relatively weak inhibitory activity against PPL as compared with other herbs such as the dried fruit-spike of *Prunella vulgaris* L. and the rhizome of *Rheum palmatum* L. [96], it could significantly suppress the activity of pancreatic lipase *in vivo*. Additionally, Raphani Semen carries fatty acids (linolenic acid [59, 60], stearic acid [61], nervonic acid [62], and palmitoleic acid [66]) which have been shown to have beneficial effect on obesity-related disorders. Therefore, Raphani Semen might have antiobesity effect.

### 4.3. Improvement of Insulin Resistance

Patients with type 2 diabetes mellitus tend to develop hypertension, which is a major cause of cardiovascular morbidity and mortality in this group of patients [97]. Although the exact mechanisms underlying this propensity remain to be clarified, insulin resistance, dysfunction, and structural abnormalities in microvascular system together with renal damage may play crucial roles in the disease progression [98]. Raphani Semen is used to remove phlegm turbidity by TCM practitioners. According to TCM theory, higher phlegm turbidity is believed to be related to increased blood viscosity [99]. In fact, increased blood viscosity is one of the inducers of insulin resistance since it could affect the normal binding capacities of the insulin receptors in vascular system. A research found that intragastric administration of Raphani Semen extract to Sprague-Dawley rats with hypertension and insulin resistance could increase the insulin-binding affinity to its receptors on hepatocytes [99]. Therefore, Raphani Semen may be effective in improving the pathologic insulin resistance state. 

### 4.4. Gastrointestinal Effect

Gastrointestinal (GI) neuromuscular diseases consist of multiple groups of chronic conditions associated with impaired gut motility. Since GI disorders (varying in etiopathogenic mechanisms, pathologic lesions, and regions of GI tract involved) are very common and are considered as one of the major social and economic burdens, they represent a relevant matter for public health [100]. Using natural products to relieve GI disorders becomes a new approach to handle these problems. It was reported that Raphani Semen water extract could enhance the contractility of smooth muscles from guinea-pig ileum [101]. Another study showed that Raphani Semen water extract exhibits significant *in vitro* GI prokinetic effects in the rabbit jejunum and ileum, rat stomach fundus, and ileum as well as the guinea-pig jejunum [102]. Additionally, Raphani Semen water extract was demonstrated to exhibit *in vivo* laxative effects in mice [102]. Interestingly, different processing methods on Raphani Semen could affect the strength of gastric emptying effect on Kunming mice. It was found that roasted Raphani Semen elicited less inhibitory effect on the gastric emptying as compared with the dried (no roasting) one [103]. Mice treated with roasted Raphani Semen had ~57% gastric residue, while mice treated with dried Raphani Semen had ~70% gastric residue [103]. In contrast, overroasted Raphani Semen could cause stomach swelling as its gastric residue content was up to 80% (the highest among the three Raphani Semen extract-treated mice) [103]. Comparing to the movement of GI smooth muscle in Kunming mice, ileum smooth muscles of rabbits fed with roasted Raphani Semen extract were much more reactive. Concerning the small intestine propulsive effect, there was no significant difference between the three Raphani Semen extract-treated mice. It could be deduced that food digestion could be enhanced better by the roasted Raphani Semen as it had the ability to retain food in the stomach longer via moderate inhibition of the gastric emptying.

### 4.5. Treatment of Constipation

Mice's intake of Raphani Semen oil in 2.4 g/kg/day, which was equivalent to the amount for human's dosage in TCM, could increase defecation rate (in terms of the number and mean weight of formed fecal pellets excreted) [104]. Additionally, same dosage of Raphani Semen water extract could shorten the defecation latency of constipation and increase the defecation rate [104]. It suggested that both Raphani Semen oil and its water extract could be effective in treating constipation.

### 4.6. Antitussive Effect

Water extract of Raphani Semen was shown to have cough inhibitory activity on Kunming mice. The antitussive therapeutic effect of the dried Raphani Semen (without roasting) was found to be much longer lasting than those of roasted and overroasted samples, while there was no significant difference in the phlegm-eliminating actions among various groups [105]. 

## 5. Toxicology

Although many positive effects of Raphani Semen were determined, different processing methods (dried, roasted and overroasted) on Raphani Semen may give different levels of toxicity. A study on the effect of processing methods on Raphani Semen's toxicity showed that Kunming mice fed with roasted Raphani Semen had the highest mortality, dirtier body hair, and more liquid-form feces, while the other two groups fed with dried and overroasted samples had relatively mild adverse responses. The observed abnormalities may be attributed to the chemical changes during the roasting process of Raphani Semen which led to the increased GI movement [103]. Another study assessed the acute toxicity of Raphani Semen cold-water extract on Balb/c mice [92]. No death of animal was observed at the maximum tested oral dose (10 g/kg). Additionally, no apparent GI or behavioral side effects were identified during the testing period [92]. The discrepancy in the results of different studies may be attributed to the difference in animal species used, content of active components in Raphani Semen extracts, and extraction methods adopted. Since different processing methods on Raphani Semen could give different levels of toxicity, more attention should be paid when choosing the processing method of Raphani Semen. It is suggested to adopt the standardized simple stir-baking method of the seeds recorded in CP (Appendix II D) [3] so as to ensure the therapeutic efficacy and reduce possible risk of inducing toxicity.

Raphani Semen contains high content of erucic acid. It is a long-chain unsaturated fatty acid. Studies found that erucic acid had adverse effect on heart as erucic acid was poorly oxidized by the mitochondrial *β*-oxidation system, especially by the myocardial cells, which resulted in an accumulation of erucic acid, producing myocardial lipidosis [106, 107]. Although the negative effect has not been confirmed in humans, a variety of animal species fed with erucic acid could result in forming myocardial lipidosis in a dose-dependent manner. Erucic acid also interacts with other drugs. When applied alone, erucic acid showed no negative effect on survival and cardiac contractility in Wistar albino rats. However, under concomitant use with doxorubicin (a drug used for chemotherapy), erucic acid could enhance the toxicity of doxorubicin [108]. So far, the adverse effects of Raphani Semen consumption due to the presence of erucic acid have not been reported. There are reports on animal studies about induction of myocardial lipidosis occurrences after the consumption of pure or high concentration of erucic acid but not the TCM herb, Raphani Semen. Owing to the multiple pharmacological effects of Raphani Semen and the high content of bioactive compounds such as linolenic acid in Raphani Semen, it is worthwhile to launch more investigations to evaluate the risks and benefits of using Raphani Semen. 

## 6. Raphani Semen in Perspectives

Despite the fact that Raphani Semen is commonly used in TCM, there is a lack of in-depth study on the pharmacological effects of its active ingredients. It was found that among the reported pharmacological effects, most of them are related to chronic diseases such as cardiovascular diseases and diabetes mellitus. For the progression of those chronic diseases, reactive oxygen species plays an important role. In view of the abundance of phenolic compounds, flavonoids, and other compounds with high antioxidant power in Raphani Semen, it may be possible to identify new drug candidate or lead compound for treating cardiovascular diseases and diabetes mellitus. Further studies on both pharmacology and chemistry could provide valuable insight into this area.

So far, there is no report about the adverse/toxicity effects of this herb on humans. However, Raphani Semen processed by roasting was reported to exhibit some adverse effects on mice. Additionally, erucic acid, an active compound from Raphani Semen, was shown to enhance the toxicity of doxorubicin. Thus, Raphani Semen has a potential risk of causing drug interaction. Since the undesirable effects of Raphani Semen overdosage are still not clearly evaluated, toxicology test is a must to examine its potential harmful effects on normal human cells and experimental animals.

In summary, Raphani Semen is a valuable TCM herb with multiple pharmacological effects. More studies on Raphani Semen should be done for a better understanding of its pharmacological mechanisms so as to provide clear scientific evidence to explain its traditional uses, to identify its therapeutic potential on other diseases, and to understand its possible harmful effects.

## Figures and Tables

**Table 1:** Bioactive compounds of Raphani Semen.

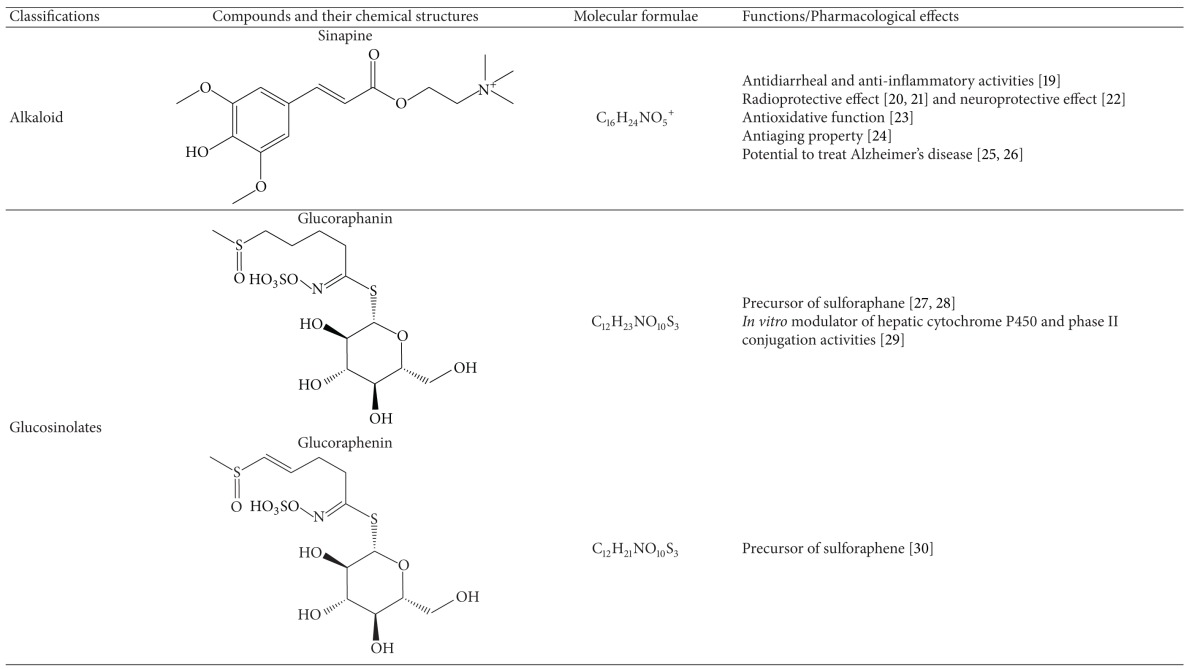 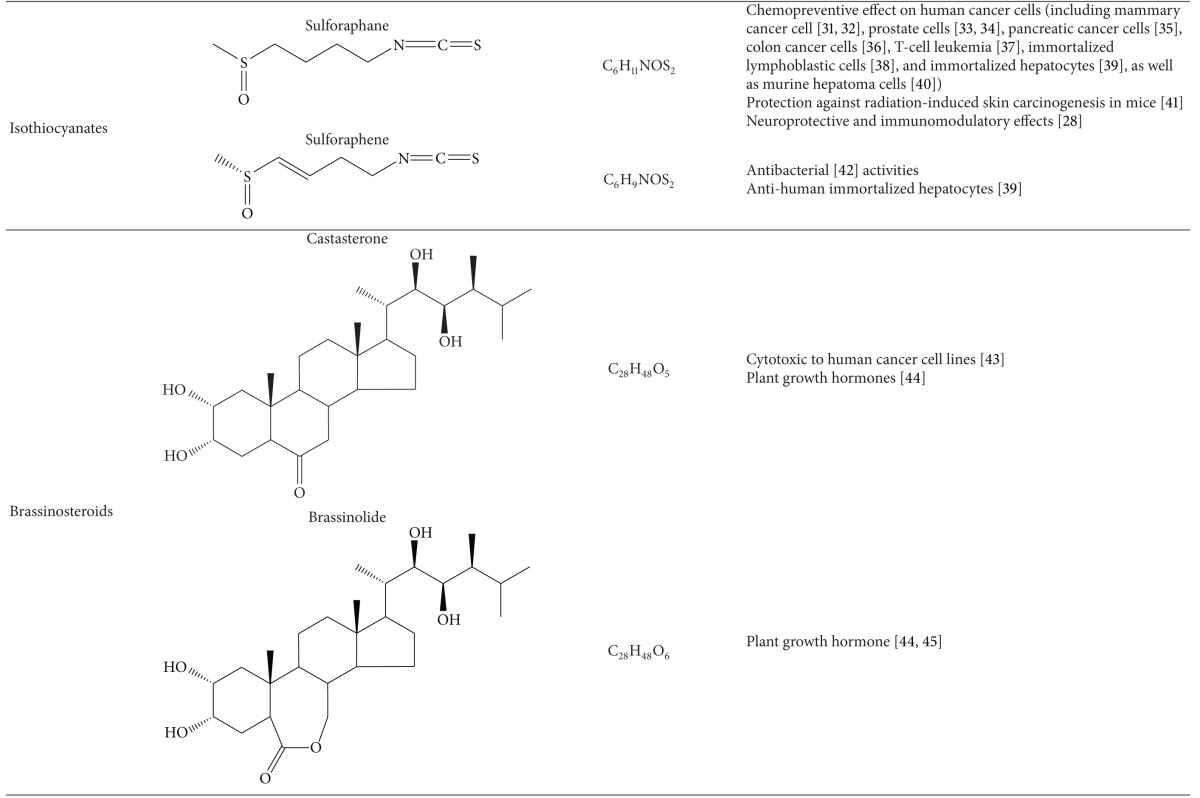 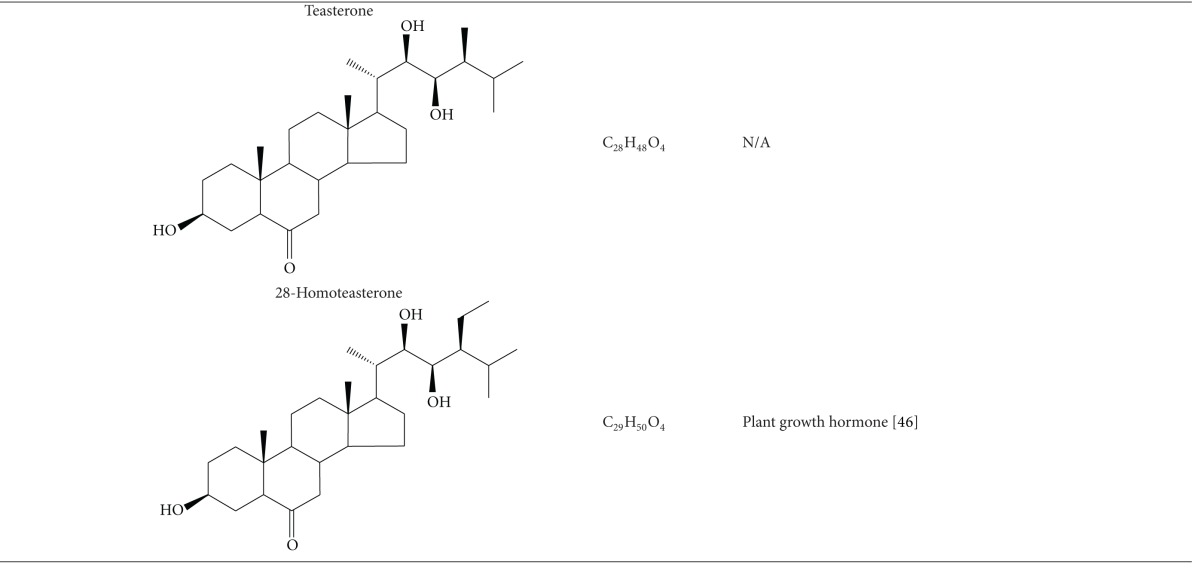

N/A; not applicable.

**Table 2:** Nutritional ingredients identified in Raphani Semen.

Classifications	Nutritional ingredients				
Fatty acids [47–51]^#^	Linolenic acid	Erucic acid	Oleic oil	Eicosenoic acid	Palmitic acid
	Stearic acid	Arachidic acid	Behenic acid	15-Tetracosanoic acid	

Tocopherol isomers [51]^##^	*α*-Tocopherol	*β*-Tocopherol	*γ*-Tocopherol	*δ*-Tocopherol	

Minerals [51]	Calcium (145 ± 4.55 *μ*g/g)	Magnesium (24.4 ± 2.67 *μ*g/g)	Iron (4.19 ± 0.311 *μ*g/g)	Zinc (1.94 ± 0.418 *μ*g/g)	

Proteins	Rs-AFP1 [52, 53]	Rs-AFP2 [52, 54]	RAP-1 [55]	RAP-2 [55]	2S albumins [52]

Polysaccharides (12.73%) [56]	L-arabino-D-galactan [57]	L-arabino-D-galactan-containing proteoglycan [57]			

^#^Details are shown in Table 3.

^##^Details are shown in Table 4.

**Table 3:** Constituents of fatty acids in Raphani Semen.

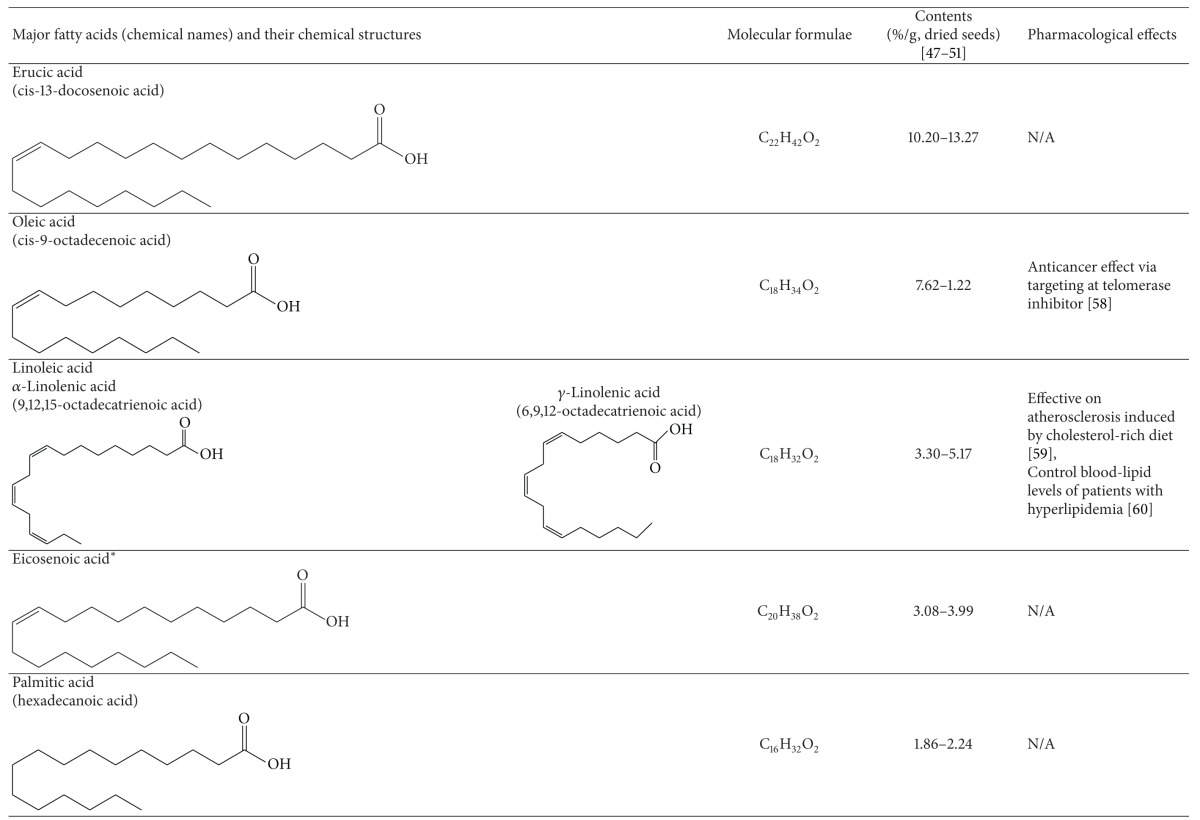 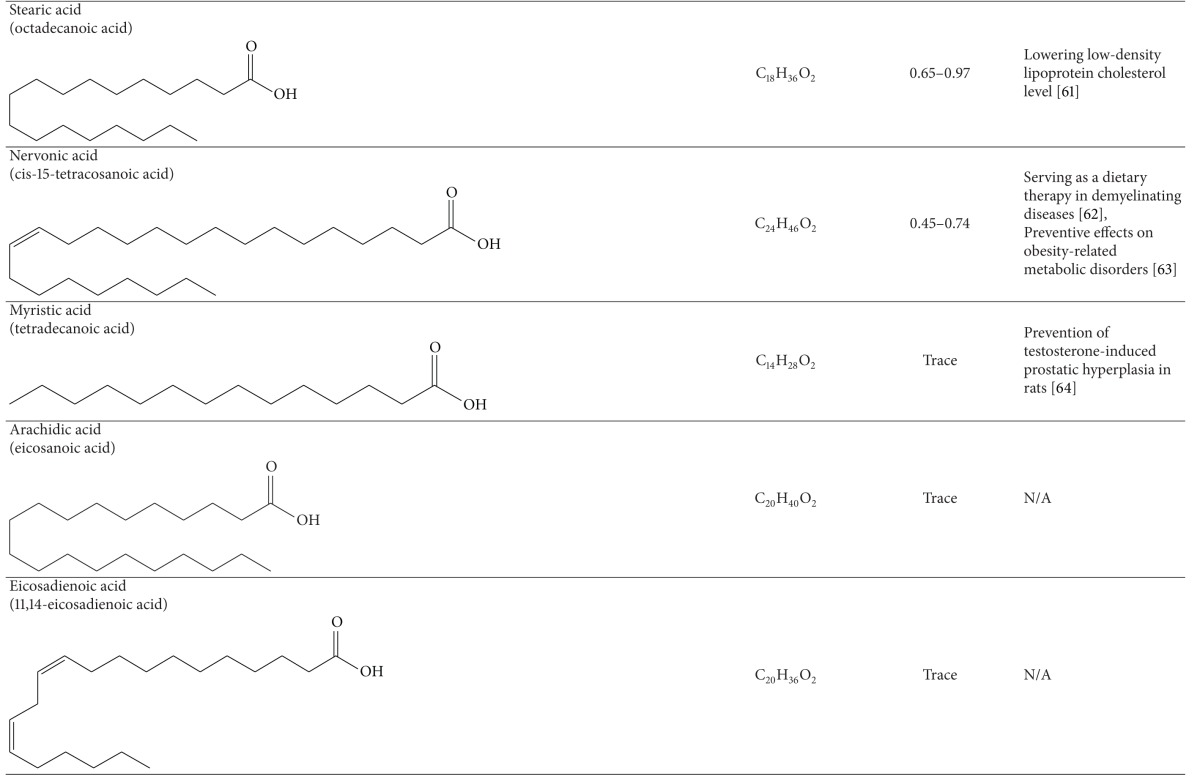 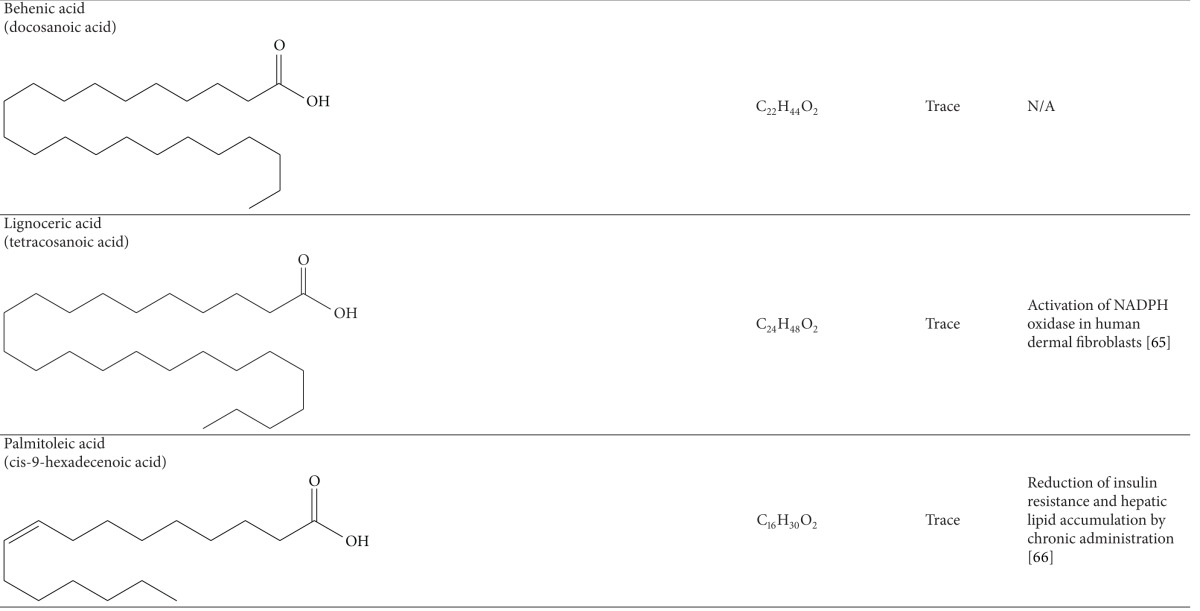

*One of the representative structures of the chemical.

N/A; not applicable.

**Table 4:** Constituents of tocopherol isomers identified in Raphani Semen [51].

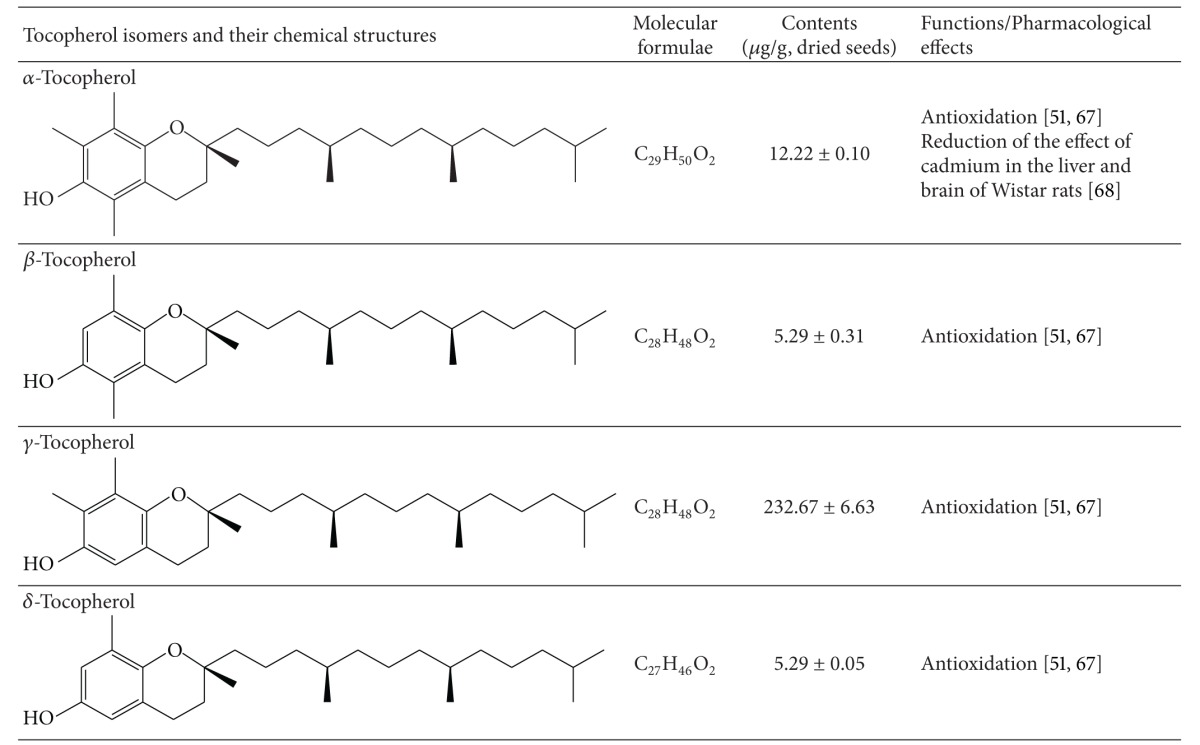

## Abbreviations

ACE: Angiotensin converting enzyme
GI: Gastrointestinal
NO: Nitric oxide
NOS: Nitric oxide synthase
CP: Pharmacopoeia of the People's Republic of China
PPL: Porcine pancreatic lipase
Rs-AFP and RAP: *Raphanus Sativus* Antifungal Protein
SHR: Spontaneously hypertensive rats
TCM: Traditional Chinese Medicine.
